# Insight into the Genome of Diverse *Penicillium chrysogenum* Strains: Specific Genes, Cluster Duplications and DNA Fragment Translocations

**DOI:** 10.3390/ijms21113936

**Published:** 2020-05-30

**Authors:** Juan F. Martín

**Affiliations:** Área de Microbiología, Departamento de Biología Molecular, Universidad de León, 24071 León, Spain; jf.martin@unileon.es

**Keywords:** *Penicillium chrysogenum*, genome sequence, strain-specific genes, gene rearrangements, gene cluster amplification, penicillin, antifungal proteins, prebiotics

## Abstract

Background: There are eighteen species within the *Penicillium* genus section *chrysogena*, including the original penicillin producers *Penicillium notatum* (Fleming strain) and *Penicillium chrysogenum* NRRL 1951. Other wild type isolates of the *Penicillium* genus are relevant for the production of useful proteins and primary or secondary metabolites. The aim of this article is to characterize strain specific genes and those genes which are involved in secondary metabolite biosynthesis, particularly the mutations that have been introduced during the β-lactams strain improvement programs. Results: The available genomes of several classical and novel *P. chrysogenum* strains have been compared. The first genome sequenced was that of the reference strain *P. chrysogenum* Wis54-1255, which derives from the wild type *P. chrysogenum* NRRL 1951; its genome size is 32.19 Mb and it encodes 12,943 proteins. Four chromosomes were resolved in *P. chrysogenum* and *P. notatum* by pulse field gel electrophoresis. The genomes of three industrial strains have a similar size but contain gene duplications and truncations; the penicillin gene cluster copy number ranges from one in the wild type to twelve in the *P. chrysogenum* ASP-E1 industrial strain and is organized in head to tail tandem repeats. The genomes of two new strains, *P. chrysogenum* KF-25, a producer of antifungal proteins isolated from a soil sample, and *P. chrysogenum* HKF2, a strain with carbohydrate-converting activities isolated from a sludge treatment plant, showed strain specific genes. Conclusions: The overall comparison of all available *P. chrysogenum* genomes indicates that there are a significant number of strain-specific genes, mutations of structural and regulatory genes, gene cluster duplications and DNA fragment translocations. This information provides important leads to improve the biosynthesis of enzymes, antifungal agents, prebiotics or different types of secondary metabolites.

## 1. Introduction: *Penicillium chrysogenum* as a Platform for the Biotechnology of Fungal Products

*Penicillium* section *chrysogena* is a large group of fungi that includes 18 *Penicillium* species [1] with important different characteristics. These species include not only the original penicillin-producing strains but also numerous building contaminant strains [2] and novel strains isolated from a variety of habitats. The discovery of penicillin by Fleming [3] has contributed drastically to improving the health of humans. In the 90 years since the discovery of penicillin, impressive progress has been made in our understanding of the biosynthesis of penicillin, and this has contributed to developing second and third generation β-lactam antibiotics.

Penicillins are modified non-ribosomal synthesized peptides that are condensed by oxidative cyclization reactions forming the penam nucleus [4,5]. Penicillin and other β-lactams are produced by several species of ascomycetes fungi [6,7,8], although they are an infrequent type of secondary metabolites; gene clusters for polyketide and lineal or cyclic non-ribosomal peptides, other than β-lactams, are more frequent in the fungal genomes. The first studies of the antibacterial activity of penicillin were made with the compound obtained from the wild type *P. notatum* [3]. Later, the search for improved penicillin producers resulted in the isolation of *Penicillium chrysogenum* NRRL1951 [9], isolated from a moldy cantaloupe in Peoria (USA). Both *P. notatum* and *P. chrysogenum* are now reclassified as *Penicillium rubens* [10], as indicated in Table 1, but the old names are kept in the text to avoid confusion, since they are maintained in most recent publications. Penicillin is also synthesized at low levels by *Aspergillus nidulans* and there have been several studies on the mechanism of the biosynthesis regulation of this antibiotic in *A. nidulans* [11,12]. *P. chrysogenum* is an excellent platform to study the production of heterologous proteins and other biotechnological compounds due to the excellent background knowledge of its molecular genetics, biochemistry and fermentation technology [13,14]. The most relevant *P. chrysogenum* strains are listed in Table 1. This article is focused on the genomes of several different strains of *P. chrysogenum* that have a wide potential for the production of antifungal proteins, prebiotics, β-lactams and other secondary metabolites. A detailed analysis of the genomes of different *P. chrysogenum* strains may provide useful information on how to direct the biosynthetic pathways for the production of new enzymes and novel secondary metabolites in those strains. This review is organized into three parts: first, the initial *P. chrysogenum* Wis54-1255 and *P. chrysogenum* P2niaD18 genomes are analyzed. The genome of the Fleming *P. notatum* strain has been sequenced (unpublished results), although some differences between the *P. notatum* and *P. chrysogenum* strains have been already reported [15,16,17]. A second section deals with the genomes of novel *P. chrysogenum* wild type strains which have not been studied taxonomically in detail (Table 1).

A third part is dedicated to discussing *P. chrysogenum* industrial strains. Large improvements in the production of penicillin from strains derived from *P. chrysogenum* NRRL1951 were obtained in the last four decades [29] by treatment with strong mutagenic agents (such as UV light, X rays, nitrogen mustard) and selection of strains with improved fermentation characteristics [19,30]. Therefore, most of the classical biochemical and genetic studies were performed in mutant strains derived from *P. chrysogenum* NRRL1951 [4,24,31].

## 2. Unveiling the Content of the Genome of *P. chrysogenum* Wis54-1255

**Figure 1:**
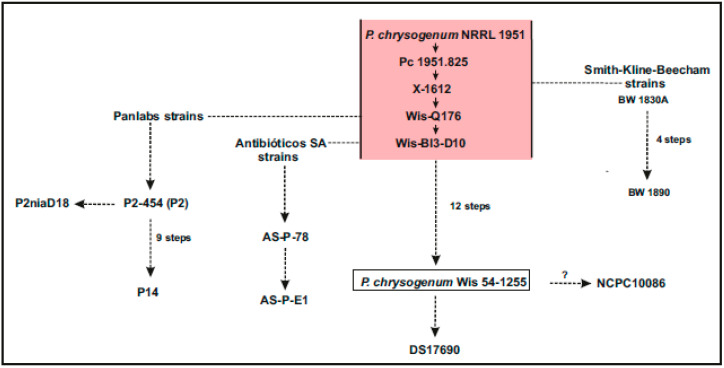
Phylogenetic three of the most relevant industrial strains of *P. chrysogenum*. This three is constructed based on the information of Lein et al. [19], Newbert et al. [22], Salo et al. [28] and Fierro et al. [24]. The number of mutagenic steps is indicated where it is known. The origin of *P. chrysogenum* NCPC10086 is discussed in the text. Stage 1 includes steps from the wild type *P. chrysogenum* NRRL1951 to *P. chrysogenum* Wis 54-1255. Stage II includes steps from *P. chrysogenum* Wis54-1255 to *P. chrysogenum* DS17690 (see text). The early steps of stage 1 from which derive some industrial lineages are highlighted in pink color, although the specific branching step is sometimes unknown. The standard reference strain *P. chrysogenum* Wis54-1255 is boxed.

Following the development of advanced next-generation genome sequencing tools, the scientists at the DSM research laboratories in Delf (Netherlands) completed the sequence of the *P. chrysogenum* Wis54-1255 genome, an improved penicillin producer strain derived from *P. chrysogenum* NRRL1951 (Figure 1). This strain had been previously studied from the biochemical and molecular genetic point of view and therefore was considered an international reference research strain. The genome of *P. chrysogenum* Wis54-1255 has a size of 32.19 Mb and was obtained by the assembling of 49 supercontigs; the GC content of the genome is 48.9% and the GC of the exons is 52.8%. Each gene contains an average of 3.0 exons and about 83.5% of the genes contain introns [32]. The number of encoded proteins was 12,943, including enzymes of at least 33 secondary metabolite gene clusters (20 PKSs, 10 NRPSs, 2 hybrid NRPS-PKSs and 1 dimethylallyltryptophan synthase). The genome sequence of the wild type strain *P. chrysogenum* NRRL1951 has not been published so far, but some DNA sequences of this strain were obtained and compared with the reference genome sequence of the improved strain *P. chrysogenum* Wis54-1255 [28]. This allowed the identification of a number of mutations that were introduced during the early steps of classical strain improvement programs (steps from the wild type *P. chrysogenum* NRRL1951 to the improved producer strain *P. chrysogenum* Wis54-1255). The results of this study showed that in parallel to the increase in penicillin production, several polyketide (PK) and non-ribosomal peptide (NRP) gene clusters were partially or even completely silenced, particularly the PKS involved in the production of the yellow pigment that is very abundant in *P. chrysogenum* NRRL1951. This pigment has been recently identified as a mixture of sorbecillinoid molecules [33]. The most relevant genome features of *P. chrysogenum* Wis54-1255 in comparison to other *P. chrysogenum* strains are shown in Table 2.

### 2.1. Reorganization of the Chromosome Architecture by DNA Fragments Translocation in the Strains P. chrysogenum Wis54-1255 and P. chrysogenum P2niaD18

Differences between the chromosome architecture in distinct strains of *P. chrysogenum* and *Acremonium chrysogenum* were observed in early studies [34,35]. The *P. chrysogenum* P2niaD18 strain derives from the Panlab strain P2 (Figure 1) and contains a *niaD* mutation that results in a nitrate reductase deficiency [36]. The genome of *P. chrysogenum* P2niaD18 was sequenced by Specht et al. [20] and contains a total of 11,749 open reading frames (Table 2). This genome was similar to that of *P. chrysogenum* Wis54-1255, both of which derived from the wild type strain *P. chrysogenum* NRRL1951. Most of these ORF were 100% identical to those of *P. chrysogenum* Wis54-1255. Interestingly, it was found that there is a duplication of the penicillin gene cluster in chromosome II of *P. chrysogenum* P2niaD18 that was not observed in the genome of *P. chrysogenum* Wis54-1255. Moreover, Specht et al. [20] found two DNA fragment translocations, one between chromosomes II and III and the other between chromosomes III and IV. The first one of these translocations caused the *niaD* mutation that resulted in nitrate reductase deficiency. Finally, Specht and co-workers [20] assembled the whole genome in four chromosomes that agree approximately in size with those reported previously by Fierro et al. [37] (see below).

### 2.2. Resolution of P. chrysogenum and P. notatum Chromosomes and Physical Map of the Encoding Genes

Classical genetics of *Penicillium* relied on the mapping of the identified genes. Pioneer studies on the resolution of four chromosomes in *P. notatum* and *P. chrysogenum* were performed by Fierro et al. [37]. Using a pulse field gel electrophoresis (PFGE) of DNA samples obtained by the direct lysis of cells in agar plugs, these authors managed to identify chromosome I (10.4 Mb), chromosome II (9.6 Mb), chromosome III (7.3 Mb) and chromosome IV (6.8 Mb). Small differences were found in the size of the four chromosomes in the five strains of *P. chrysogenum,* particularly in the penicillin high producing strain ASP-78. The total genome size estimated by PFGE is 34.1 Mb for the *P. chrysogenum* strains, which roughly agrees with the deduced genome size after genome sequencing [32]. The complete penicillin gene cluster was found by hybridization with probes internal to the *pen* cluster and mapped in chromosome I in *P. chrysogenum* Wis54-1255. Other genes were found to be located in other chromosomes, e.g., the *pyrG* gene was located in chromosome II in all *P. chrysogenum* strains except in strain ASP-78, in which it was located in chromosome III, indicating that a translocation had occurred in this strain [35]. In a comparative study of *P. chrysogenum* and the *P. notatum* Fleming strain, it was found that *P. notatum* has four chromosomes of 10.8, 9.6, 8.3 and 5.4 Mb (Table 3) [37]. The *P. notatum* genome size was estimated to be 32.1 Mb, slightly smaller than that of *P. chrysogenum* Wis54-1255. Interestingly, the penicillin gene cluster of *P. notatum* maps in chromosome II, in contrast to what was found in *P. chrysogenum*. These differences may be explained by divergent evolution in the last centuries in the distinct habitats from which they were isolated; the Fleming strain is considered a building indoor contaminant strain as other air-borne *Penicillium* species, whereas the *P. chrysogenum* Wis54-1255 strain derives from *P. chrysogenum* NRRL1951, isolated from a moldy cantaloupe [2].

An improvement of the physical map was proposed by Xu et al. [38] using the insertion of large *P. chrysogenum* fragments in binary artificial bacterial chromosomes, but the results of this technique was not exploited in detail. The availability of the full genome sequence in *P. chrysogenum* Wis54-1255 and *P. chrysogenum* P2niaD18 [20,32], both derived from *P. chrysogenum* NRRL1951, allowed researchers to find 179 gaps existing in the genome of *P. chrysogenum* Wis54-1255. This resulted in the assembly of four chromosomes’ physical maps and one circular mitochondrial DNA. The exact sizes of the four chromosomes of these two strains are given in Table 3. Some differences in the size of the chromosome of *P. chrysogenum* P2niaD18 with respect to the *P. chrysogenum* Wis54-1255 strain are due to translocations between chromosomes II and III and chromosomes III and IV, as described above. 

### 2.3. The Genome Sequence of the Wild Type Isolate P. chrysogenum Pc3 Contains Numerous Differences with Respect to P. chrysogenum NRRL1951

During a study of the conjugation between the two different mating type strains of *P. chrysogenum*, the genome of a novel wild type soil isolate, *P. chrysogenum* Pc3, was sequenced [21]. This strain is particularly relevant because it contains the mating type MAT1-2 and therefore is able to conjugate with *P. chrysogenum* NRRL1951 and its derivatives, which show the MAT1-1 mating type.

The genome size of this strain is 32.2 Mb and it encodes 11,198 proteins. This strain produces very low levels of penicillin and contains a single copy of the penicillin gene cluster. Interestingly, the genome sequence of the *P. chrysogenum* Pc3 revealed that it contains thousands of single nucleotides polymorphisms (SNP) in numerous genes when compared with the genome of the *P. chrysogenum* P2niaD18 strain, confirming that these two strains are distantly related.

The nucleotide sequences of the four chromosomes were studied and their sizes in *P. chrysogenum* Wis54-1255 were slightly different from those of the *P. chrysogenum* P2niaD18 strain [20,21] (Table 3). The alignment of the nucleotide sequences of *P. chrysogenum* Pc3 with that of the reference *P. chrysogenum* P2niaD18 strain showed two rearrangements in contigs 24 and 35; these two contigs of strain *P. chrysogenum* Pc3 are located in the opposite end of the respective chromosome in *P. chrysogenum* P2niaD18.

The comparative studies of the chromosome sizes of several strains such as *P. chrysogenum* ASP-78 allowed researchers to conclude that there are significant differences in the architecture of the chromosomes due to DNA fragment translocations [35].

## 3. Genomes of Other Wild Type *P. chrysogenum* Strains

In addition to the classical penicillin-producing strains, *P. notatum* (Fleming strain) and *P. chrysogenum* NRRL1951 and its derivatives, in recent years a growing interest has developed in the identification and characterization of wild type *P. chrysogenum* strains relevant for the production of antifungal agents, prebiotics and extracellular enzymes.

### 3.1. The Genome of the Wild Type P. chrysogenum KF-25 and Differences with P. chrysogenum Wis 54-1255

*P. chrysogenum* strains have important roles in the production of antifungal agents and plant growth elicitors and in the obtention of extracellular hydrolytic enzymes [13]. Many *P. chrysogenum* strains produce penicillin, but some others may lack the penicillin gene cluster and therefore they may be useful in searching for new types of metabolites that are not masked by the production of penicillin [39,40,41]. Particularly relevant is the production of small proteins with antifungal activity, such as the Paf and Gaf proteins produced by *P. chrysogenum* and other ascomycetes [42,43]. These small proteins are antifungal agents used in the control of fungal infections produced by other filamentous fungi [44,45]. *P. chrysogenum* KF-25 was isolated from a soil sample in China and selected for its activity against *Ustilaginoidea virens* infections [25].

The genome of *P. chrysogenum* KF-25 was obtained in 194 scaffolds with an average size of 97.15 Kb. This genome encoded 9804 ORFs and is 2300 Kb smaller than that of *P. chrysogenum* Wis54-1255 (Table 2). The large difference in genome size between these two strains is intriguing but not unusual, taking into account the different geographical regions and the material from which they were isolated. Therefore, a relevant question is which are the genes of *P. chrysogenum* Wis54-1255 that are missing in *P. chrysogenum* KF-25, or vice versa? An analysis of the proteins encoded in the *P. chrysogenum* KF-25 genome revealed that 71% of the proteins are orthologous to those encoded by *P. chrysogenum* Wis54-1255 genes. The missing genes in the *P. chrysogenum* KF-25 strain correspond to genes located in the 5′ and 3′ ends of the *P. chrysogenum* Wis54-1255 supercontigs, and they are also absent from the genomes of other *Penicillium* species. This indicates that these genes are strain-specific genes [3]. Some of them may correspond to secondary metabolites gene clusters that may have been acquired or lost by horizontal gene transfer [8]. Some other *P. chrysogenum* Wis54-1255 strain-specific genes correspond to proteins of unknown function which frequently are found in repetitive sequences, or to virus or transposons-related sequences [25,46]. It is noteworthy that in *P. chrysogenum* KF-25 there are 355 specific genes that are absent from *P. chrysogenum* Wis54-1255. Most of these genes are similar to genes occurring in species of *Aspergillus* or *Neosartoria,* suggesting that they may derive from a common ancestor of all these fungi. Some of the *P. chrysogenum* KF-25 strain-specific genes include genes involved in protein secretion, intracellular compartmentalization, vesicular traffic and regulatory mechanisms [25] that play important roles in protein secretion—e.g., in the secretion of extracellular antifungal proteins.

Regarding the pigmentation and production of secondary metabolites, the *P. chrysogenum* KF-25 strain is more highly yellow pigmented than *P. chrysogenum* Wis54-1255, and in this respect is similar to the original *P. chrysogenum* NRRL1951 isolate [9], suggesting that this strain has a more intense production of sorbecillinoid pigments. Thirty-three putative secondary metabolites encoding gene clusters were found in *P. chrysogenum* KF-25 compared to forty-one found in *P. chrysogenum* Wis54-1255. These secondary metabolites (SM) gene clusters include 8 clusters encoding NRPSs, 10 clusters encoding PKSs, 2 clusters for hybrid NRPS-PKSs, 1 cluster encoding a putative terpene, 1 cluster for a hybrid PKS-terpene, 1 cluster for a siderophore and 10 additional clusters for complex poorly characterized metabolites. Some of the putative SMs encoded by these clusters coincide with those described in *P. chrysogenum* Wis54-1255 [14]. The penicillin gene cluster of *P. chrysogenum* KF-25 was found in a single copy, as occurs in *P. chrysogenum* NRRL1951 and *P. chrysogenum* Wis54-1255. Finally, the genome of *P. chrysogenum* KF-25 reveals that it encodes a number of proteins involved in pathogenicity to plants that are similar to those found in strains derived from the wild type strain *P. chrysogenum* NRRL1951 [13,26].

### 3.2. The Genome of P. chrysogenum HKF2: Potential for the Production of Prebiotics

The ability of *Penicillium* to degrade carbohydrates due to its secretion of carbohydrate-converting enzymes is well known [13,26]. These enzymes have great potential for the production of prebiotics. These compounds are defined as non-digestible food ingredients that promote the growth of beneficial bacterial in the human and animal gut, and they may be used to improve the health of the human beings [47,48]. Prebiotics include several types of carbohydrates, such as isomaltose-oligosaccharides, galacto-oligosaccharides, inulo-oligosaccharides, fructo-oligosaccharides and chito-oligosaccharides. During a search for strains able to produce carbohydrate-modifying enzymes for prebiotics production, Gujar et al. [26] isolated the strain *P. chrysogenum* HKF2 from the activated sludge of a plant for waste treatments. The draft genome sequence of this strain reveals that it consists of 31.5 Mb, encoding 11,243 genes. Six hundred and nine of these genes encode carbohydrate active enzymes closely related to those encoded by *P. chrysogenum* strains and to some extent to other *Penicillium* species. These authors focused their work on the isolation of genes encoding some of the more relevant carbohydrate active enzymes, but they have not studied the genes for secondary metabolism of *P. chrysogenum* HKF2. Some of the most relevant enzymes include exo-inulinase and β-fructofuranosidase, which have potential for the formation of inulinin oligosaccharides and fructose oligosaccharides prebiotics. Since this strain was isolated from a habitat completely different from the soil, it would be very interesting to study the genes for secondary metabolism in *P. chrysogenum* HKF2.

## 4. Enigmas in the Genome of Industrial High Penicillin-Producing Strains

Several pharmaceutical companies have developed high penicillin-producing strains (Figure 1). An enigmatic point that remains obscure is how increases higher than one thousand-fold in penicillin production have been achieved in these overproducing strains.

However, most of the information about the mutations introduced in these strains is proprietary and frequently it is not even known which mutations have been introduced. We summarize in this section the information available on strains developed in: (1) Antibióticos SA (León, Spain), (2) Smith Kline Beecham laboratories (United Kingdom), (3) Biotika AS (Slovaquia), (4) DSM laboratories (Delf, The Netherlands) and (5) The North China Pharmaceutical Corporation (China).

The characterization of the high penicillin-producing strains *P. chrysogenum* ASP-78 and *P. chrysogenum* ASP-E1, provided by Antibióticos SA; the *P. chrysogenum* BW 1890 strain of Smith Kline Beecham; and the Biotika AS strains focuses on the analysis of the amplified regions that occur in these high penicillin-producing strains [22,23,24]. The other three industrial laboratories described above have provided the complete genome sequences of strains *P. chrysogenum* P2niaD18 [20], *P. chrysogenum* DS17690 [28] and *P. chrysogenum* NCPC10086 [27] (see Table 1).

Early studies on the characterization of penicillin high-producing strains revealed that the genes *pcbC* and *penDE* were amplified in several copies in distinct *P. chrysogenum* strains [49]; this was later extended to the three genes that encode the biosynthetic enzymes δ-(L-α-aminoadipyl-L cysteinyl-D-valine) (ACV) synthetase, isopenicillin N (IPN) synthase (cyclase) and IPN acyl transferase [50]. A detailed analysis of the DNA region that encodes the penicillin gene cluster and adjacent regions [24] established that a region of 106.5 Kb in strain *P. chrysogenum* ASP-78 and 57.9 in *P. chrysogenum* ASP-E1 is amplified head to tail in tandem repeats in those strains. The amplified region contains 5-6 copies in *P. chrysogenum* ASP-78 and 12-14 in *P. chrysogenum* ASP-E1 [24], and the tandem repeat copies are linked by a conserved hexanucleotide TTTACA sequence. It is interesting that strains obtained in different laboratories that lacked the production of penicillin have lost the entire penicillin gene cluster by the deletion of the amplifiable region at the border hexanucleotide TTTACA sequence [31,51]. Later, Newbert et al. [22] studied the amplification of the penicillin cluster in the Smith Kline Beecham series of improved strains and observed amplifications ranging from one copy (strain BW1900A) to 50 copies in *P. chrysogenum* BW1952. The 50 copies amplification is surprising, since in other industrial strains such as *P. chrysogenum* ASP-E1 the number of copies of the amplified regions is 12–14 and 8 in the *P. chrysogenum* DS17690 strain. It is unknown if this large number of copies in the strain *P. chrysogenum* BW1952 corresponds to the tandem amplification of the whole 57.4 Kb penicillin cluster region or it has occurred by another recombination mechanism. Indeed, there is no strict correlation between the number of copies present in these strains and the penicillin levels produced. Newbert et al. [22] confirmed the organization of the amplified region described by Fierro et al. [24] and observed that there were no differences in the sequences of the promoter regions of the penicillin cluster genes. The two strains of Biotika AS, *P. chrysogenum* NMU2/40 and *P. chrysogenum* B14, were found to contain four and six copies of the entire penicillin gene cluster, respectively [23].

In conclusion, all the available information indicates that there are no differences in the promoter regions of penicillin cluster genes that may explain the differences in penicillin production. Therefore, mutations in genes outside of the amplified region probably are the reason for the distinct levels of expression of the *pen* gene cluster in the various industrial strains (see below).

### 4.1. Mutations and Translocations in the Genomes of two High Penicillin Producing Strains Result in Drastic Changes in Gene Expression

The genomes of two industrial strains, *P. chrysogenum* DS17690 and *P. chrysogenum* NCPC10086, are available. The analysis of the differences of their genomes with respect to that of the standard reference strain *P. chrysogenum* Wis54-1255 provides interesting information about the genes that affect the biosynthesis of penicillin and other secondary metabolites. In addition, as indicated above, the genome sequences of an improved strain *P. chrysogenum* P2niaD18 that is an intermediate in the Panlabs strain improvement program are available. All these three strains derive from the wild type *P. chrysogenum* NRRL1951 (Figure 1).

The comparative analysis of these genomes reveals that a large number of mutations has been introduced to obtain the current *P*. *chrysogenum* industrial strains. For simplicity, this high number of mutations was grouped in two sets of alterations (stages I and II)—the first stage resulted in the conversion of the wild type *P. chrysogenum* NRRL1951 strain to the reference laboratory strain *P. chrysogenum* Wis54-1255; the second stage included mutations that converted the strains of *P. chrysogenum* Wis54-1255 to the *P. chrysogenum* DS17690 strain [28] (Figure 1). Nine out of 31 genes (11 for NRPSs and 20 for PKSs) were found to accumulate mutations during both stages of the classical strain improvement program. Relevant mutations are those affecting the gene encoding the partially reducing PKS2 that has similarity to a diketide synthase encoded by the lovastatin *lovF* gene. The mutation that occurs in the keto synthase domain of the PKS2 probably has inactivated this enzyme. The modification of the relative abundance of the proteins encoded by the *lovC* and *lovD* genes was also found in other high penicillin-producing strains [52]. Also, non-sinonimous mutations were introduced during the second stage of the strain improvement program in the PKS7 and PKS8 genes that encode still-uncharacterized polyketide compounds. A mutation affecting a major facilitator superfamily (MSF) transporter was also acquired in stage two, but the role of this transporter in secondary metabolite secretion has not been studied so far. The mutated transporter gene is linked to the PKS18 gene, encoding the 6-methylsalicylic acid synthetase that was annotated to be involved in the synthesis of patulin, although this mycotoxin has not been detected to be produced in *P. chrysogenum* due to the lack of the genes encoding the isoepoxidon dehydrogenase, which catalyzes a middle step in the patulin biosynthetic pathway. However, the 6-methylsalicylic acid intermediate may be a precursor of other secondary metabolites, e.g., yanuthone [40]. An important mutation in stage II affects the number of copies of the penicillin gene cluster, which has gained seven additional copies in *P. chrysogenum* DS17690 [28] as compared to one copy in the *P. chrysogenum* Wis54-1255 strain. Important mutations were also found in the *laeA* and *velA* genes in the high-producing *P. chrysogenum* DS17690 strain. The effect of these mutations has been studied [53,54] and reviewed by Martín [55] and is not detailed here.

### 4.2. The Genome of the Industrial Strain P. chrysogenum NCPC10086 Shows Small Differences with Other Industrial Strains

The comparative analysis of the genomes and transcriptomes of different industrial strains developed by distinct pharmaceutical companies provides interesting information on how those strains have reached impressive penicillin production levels. The genome of a different strain, *P. chrysogenum* NCPC10086, described as an industrial strain of the North China Pharmaceutical Group Corporation, was published by Wang et al. [27] (Table 1). The origin and genealogy of this strain was not included in the article, but the authors indicate that this strain was phylogenetically close to *P. chrysogenum* Wis54-1255. Therefore, it is likely that these strain, as other industrial strains, derived from the wild type *P. chrysogenum* NRRL1951, although it was not clear whether this industrial strain derived from *P. chrysogenum* Wis54-1255 or from an earlier strain in the classical improvement program. The size of the genome was found to be 32.3 Mb with a GC content of 48.9%, similar to that of *P. chrysogenum* Wis54-1255 (Table 2). After the initial searching of the open reading frames in the genome, the authors completed the identification by comparison with the *P. chrysogenum* Wis54-1255 genome and identified 13,290 ORFs. Most of the identified genes were 100% identical in these two strains or showed small differences that may correspond either to single point mutations or to sequencing ambiguities. The authors redefined ORFs described previously as not coding regions in *P. chrysogenum* Wis 54-1255. Interestingly, Wang et al. [27] reported 69 new genes that were not found in the genome of *P. chrysogenum* Wis54-1255; perhaps most of these novel genes correspond to DNA sequences missed in the assembly of the scaffolds in the *P. chrysogenum* Wis54-1255 strain. These new genes are involved in aminosugar metabolism, nucleotide metabolism, glycogen metabolism and oxidative phosphorylation, which may result in an improved energy availability. Two additional novel genes are involved: (1) in nitrogen metabolism, encoded by a gene that is strongly induced by phenylacetic acid and may have an impact on penicillin biosynthesis; and (2) a gene involved in glutathione reduction that may affect the formation of the reduced form of the tripeptide ACV [56,57].

The three structural penicillin biosynthetic genes are linked in the *P. chrysogenum* NCPC10086 genome in 8 copies as compared to 5–6 copies in the ASP-78 strain, 12–14 in the ASP-E1 strain [24] and also 8 copies in the strain *P. chrysogenum* DS17690 [28]. The organization of the repeated copies of the *pen* gene cluster in NCPC10086 is similar to that observed in *P. chrysogenum* ASP-78 [24], except that one of the units appears to be positioned in the opposite orientation. Each copy has 56.9 Kb and is linked in tandem repeats by an hexanucleotide sequence, as described by Fierro et al. [24,31]. All this information suggests that there is a close genealogy relationship between the ASP-78 strain and the *P. chrysogenum* NCPC10086 strain. In addition, two large translocations occurred during the last steps of the *P. chrysogenum* NCPC10086 strain improvement program as compared to *P. chrysogenum* Wis54-1255. The first of these large translocations includes a 266 Kb fragment, encoding 107 genes that has moved from the subtelomeric region in *P. chrysogenum* Wis54-1255 to a centromeric region in the same chromosome [27]. This entire region is internal to scaffold Pc22g of *P. chrysogenum* Wis54-1255. The translocation has been confirmed by sequencing the borders of the integrated sequences in the new position of the genome. The translocated fragment included the nitrogen regulation gene *nre* and the authors suggested that the expression of *nre* is modified due to the translocation, thus affecting nitrogen metabolism in the *P. chrysogenum* NCPC10086 strain. A second large translocation has resulted in the transfer of a 1202 Kb fragment that is located in a different position in *P. chrysogenum* Wis54-1255 and *P. chrysogenum* NCPC10086; this fragment contains genes for a ATP/ADP mitochondrial carrier protein that is involved in energy metabolism and also the *pex2* gene that encodes a peroxin involved in the peroxisomes structure. This translocation may affect the expression of those genes and therefore may impact energy metabolism and peroxisome formation [27]. Additionally, by comparing in the strains *P. chrysogenum* Wis54-1255 and *P. chrysogenum* NCPC10086 the genes involved in phenylacetic acid hydroxylation, that degrade this precursor of penicillin biosynthesis, these authors found that the *pahA* gene is identical in both strains, including the distinct amino acid sequence reported by Rodríguez-Sáiz et al. [15] that is different in the wild type *P. chrysogenum* NRRL1951 with respect to the *P. notatum* Fleming strain. This finding supports the conclusion that the *P. chrysogenum* NCPC10086 derived from the wild type *P. chrysogenum* NRRL1951, although it cannot be excluded that wild type strains other than *P. chrysogenum* NRRL1951 also contain the same *pahA* characteristic sequences. Moreover, the *pahA* gene is identical in both strains and is present in a 27.6 Kb translocated fragment in *P. chrysogenum* NCPC10086. This raises the question of whether this translocation of the *pahA* gene affects the expression of this gene but this has not been clarified at present [27]. Three other phenylacetate hydroxylase-encoding genes related to *pahA* (*pahB*, *C* and *D*) were also studied, and the authors found that there were synonymous and non-synonymous mutations in *pahC* and *pahD*. Finally, the *P. chrysogenum laeA* gene was found to be identical in both strains. Regarding the *P. chrysogenum velA* gene, the authors found that there are some synonymous mutations and one truncation at Gln^315^ to a stop codon in this gene. Interestingly, the same truncation that reduces the size of VelA from 562 to 315 amino acids was also reported in the *P. chrysogenum* DS17690 strain (introduced during the second stage of the strain improvement program), supporting the conclusion that the *P. chrysogenum* industrial strains of DSM and North China Pharmaceutical Group Corporation have a closely related genealogy. Overall, the result of the comparison of the gene sequences of *P. chrysogenum* Wis54-1255 and *P. chrysogenum* NCPC10086 indicate that this industrial strain most likely derives from *P. chrysogenum* Wis54-1255 and is related in its genealogy to the *P. chrysogenum* DS17690 high penicillin-producing strain (Figure 1). In summary, the analysis of the genome of strain *P. chrysogenum* NCPC10086 indicates that there are mutations affecting energy metabolism, nitrogen regulation and glutathione reduction that may impact on penicillin biosynthesis, but more detailed studies are required to clarify the quantitative effect of each of these mutations in penicillin biosynthesis. These studies require directed mutations affecting these and other candidate genes that control penicillin biosynthesis in the genetic background of selected strains that already have a favorable energy and intermediary metabolism for penicillin biosynthesis.

## 5. Concluding Remarks

The recent availability of the genomes of *P. chrysogenum* KF-25 isolated from a soil sample in China and *P. chrysogenum* HKF2 isolated from a sludge plant treatment [25,26] provide new insight into the characteristics of non-classical *P. chrysogenum* strains that may lead to the discovery of new compounds relevant in: (1) human health, such as prebiotics; (2) in agriculture and plant protection [25]; or (3) new molecules with interesting pharmacological properties, e.g., antifungal proteins [58]. Only a limited amount of information on the content of these new *P. chrysogenum* genomes has been published, but the availability of the complete genome sequences will allow to mine genes encoding useful biosynthetic gene clusters and/or novel enzymes that may enlarge the scope of *P. chrysogenum* as a broad platform for the production of biotechnological products [59]. One example is the production of compactin in strains of *P. chrysogenum* [60], introducing the compactin gene cluster from *Penicillium citrinum*. A further refinement of this procedure was achieved by expressing in the compactin-producing *P. chrysogenum* transformant a P450 monooxygenase-encoding gene that hydroxylates compactin at carbon C-6, converting this compound to pravastatin [60]. On the other hand, the large amount of information on the molecular genetics of *P. chrysogenum* NRRL1951 and derived strains [61] allows us to understand which molecular mechanisms have been altered in the penicillin-producing strains, which provide useful information for directed mutations to change the profile of secondary metabolites produced by these strains. For example, the finding that several mutations have been introduced during the classical strain improvement programs in *P. chrysogenum* NRRL1951 on the LaeA and VelA-encoding genes will allow researchers to introduce the same mutations in other strains or even in other filamentous fungi to change the expression of secondary metabolite gene clusters that at present are produced at very low levels [54,62]. The CRISPR-based advances in the gene inactivation and deletion methods of manipulation in *P. chrysogenum* NRRL1951 [63] allow the exploitation of directed changes in the genome to construct a new generation of secondary metabolite-producing strains. However, in spite of the amount of molecular genetic information available, still there are many obscure points in the mechanisms of regulation at the transcriptional, translational and post-translational levels that need to be investigated.

## Figures and Tables

**Table 1:** *Penicillium chrysogenum* strains studied by different research groups.

Strain	Copies of the Penicillin Gene Cluster	Source and Reference
P. notatum *	1	[3]
*P. chrysogenum* NRRL1951 *	1	[9]
*P. chrysogenum* Wis54-1255 *	1	[18]
*P. chrysogenum* P2 *	2	[19]
*P. chrysogenum* P2niaD18 *	2	[20]
*P. chrysogenum* Pc3	1	[21]
*P. chrysogenum* BW series *	Up to 50	Beecham Corp, UK [22]
*P. chrysogenum* NMU2/40 *	4	Biotika AS [23]
*P. chrysogenum* B14 *	6	Biotika AS, Slovakia [23]
*P. chrysogenum* WE1	1	Inst. of Mol. Biol., Slovak Acad. Sci., Bratislava [23]
*P. chrysogenum* ASP-78 *	5–6	Antibióticos SA, Spain [24]
*P. chrysogenum* ASP-E1 *	12–14	Antibióticos SA, Spain [24]
*P. chrysogenum* KF25	1	[25]
*P. chrysogenum* HKF2	unknown	[26]
*P. chrysogenum* NCPC10086 *	8	[27]
*P. chrysogenum* DS17690 *	8	[28]

The strains labelled with an asterisk are now reclassified as *Penicillium rubens*.

**Table 2:** Molecular characteristics of the genomes of sequenced *P. chrysogenum* strains.

Characteristics	*Penicillium chrysogenum strain*					
	Wis54-1255	KF-25	HKF2	NCPC10086	P2niaD18	Pc3
Genome size (Mb)	32.2	29.92	31.48	32.2	32.49	32.2
GC content (%)	48.9	49.0	53.21	48.9	NA	49
Gene number	12943	9804	11243	13290	11839	11460
Average gene size (pb)	1.515	1.573	NA	1.499	NA	NA
Introns/gene	2.2	2.2	NA	NA	NA	NA
Exons/gene	3.0	3.2	3.15	NA	NA	NA
tRNA (Number)	145	112	188	NA	188	213
rRNA (number)	28	29	NA	NA	NA	49
Accession Number	AM920416 AM920464	SRP022930	MUXA00000000	APKG00000000	JMSF00000000	JPDR00000000

NA: Not available.

**Table 3:** Size of individual chromosomes in *P. chrysogenum* and *P. notatum* strains.

Strain	Chromosome I	Chromosome II	Chromosome III	Chromosome IV	Size Determination Method	Reference
*P. notatum*	10.8 Mb	9.6 Mb	6.3 Mb	5.4 Mb	PFGE	[37]
*P. chrysogenum* NRRL 1951	10.4 Mb	9.6 Mb	7.3 Mb	6.8 Mb	PFGE	[37]
*P. chrysogenum* Wis-54-1255	10.3 Mb	9.7 Mb	7.2 Mb	6.3 Mb	PFGE	[37]
*P. chrysogenum* Wis-54-1255	10,350,089 bp	9,488,591 bp	6,943,310 bp	5,586,572 bp	Sequencing	[32,20]
*P. chrysogenum* P2niaD18	13,597,116	10,455,537	5,401,030	3,043,715	Sequencing	[20]

## Abbreviations

SM	secondary metabolites
PK	polyketide
NRP	non-ribosomal peptide
PKS	polyketide synthase
NRPS	non-ribosomal peptide synthetase
ACV	δ-(l-α-aminoadipyl-l cysteinyl-d-valine)
ORF	open reading frame
SNP	single nucleotide polymorphism
CRISPR	Clustered Regularly Interspaced Short Palindromic Repeats
